# Supplementary material for: PEG-BHD1028 Peptide Regulates Insulin Resistance and Fatty Acid β-Oxidation, and Mitochondrial Biogenesis by Binding to Two Heterogeneous Binding Sites of Adiponectin Receptors, AdipoR1 and AdipoR2
Source: Int J Mol Sci. 2021 Jan 17;22(2):884. doi: 10.3390/ijms22020884 (PMC7830917; doi:10.3390/ijms22020884)
Supplement: Supplementary file 1 [file ijms-22-00884-s001.pdf]

SUPPLEMENTAL FIGURES AND TABLES

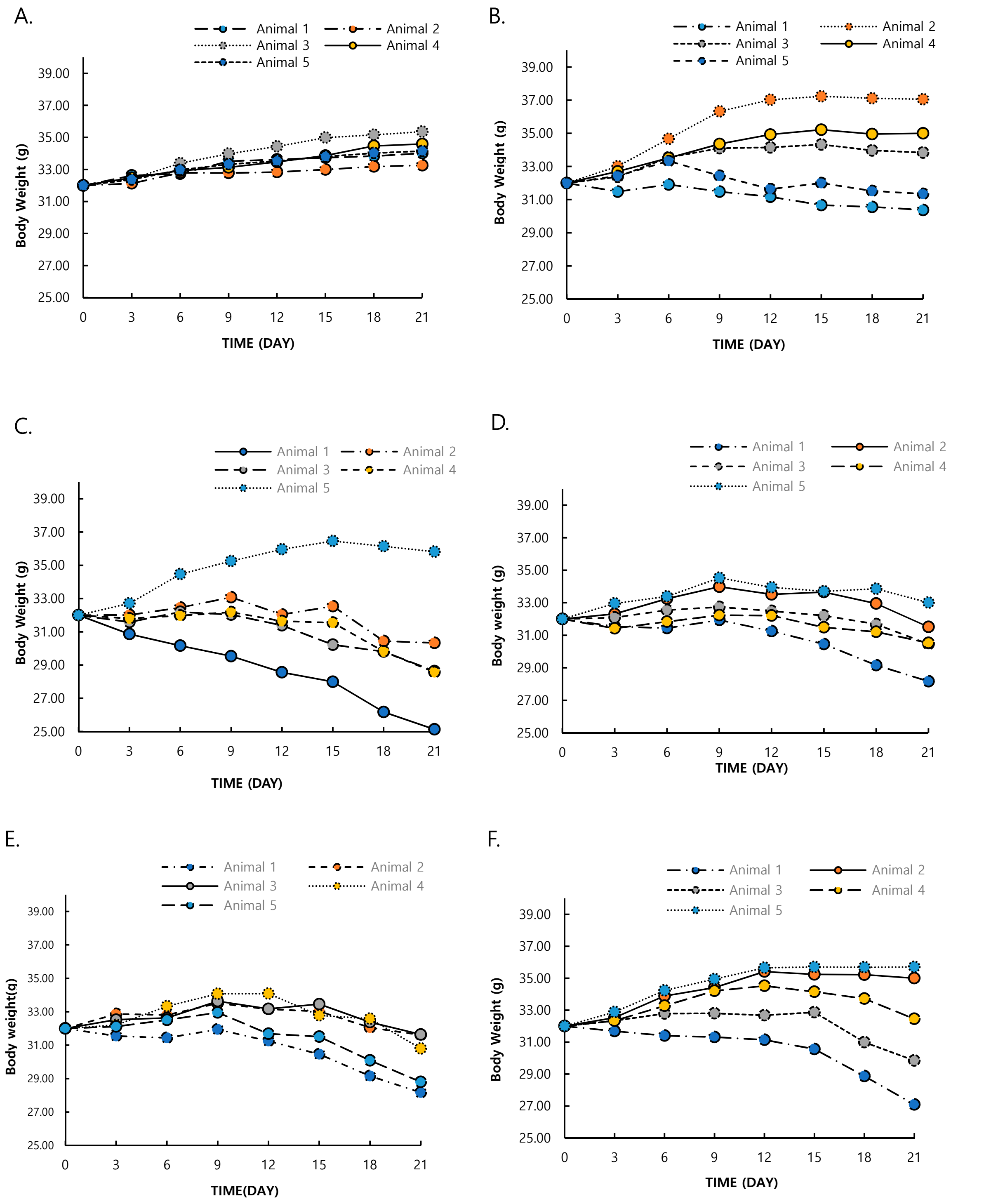

Supplemental Figure 1. Weight changes of each animal per group during the testing period (21 days). A. vehicle group, B. 1 μg/Kg group, C. 5 μg/Kg group, D. 25 μg/Kg group, E. 50 μg/Kg group, and F. 200 μg/Kg group. Each data point was normalized at 32 g.

Supplemental Table 1. Average food consumption during the testing period (n=5 per group). Each data point represents the mean food consumption of five animals ± S.E.

Group \ Day	**1**	**3**	**6**	**9**	**12**	**15**	**18**	**21**	**Ave.**
Vehicle (g)	5.70 ±0.49	9.09 ±0.33	9.23 ±0.53	9.86 ±0.37	9.84 ±0.26	9.33 ±0.64	8.58 ±0.71	8.98 ±0.74	8.83 ±0.51
PEG-BHD1028 1ug/Kg (g)	5.46 ±0.22	8.52 ±0.34	9.00 ±0.14	8.89 ±0.99	8.98 ±0.60	8.58 ±0.42	7.73 ±0.14	8.48 ±0.58	8.21 ±0.43
PEG-BHD1028 5ug/Kg (g)	5.76 ±0.30	7.67 ±0.73	7.35 ±0.72	8.31 ±0.51	7.99 ±0.42	7.99 ±0.59	6.96 ±0.54	8.09 ±0.66	7.51 ±0.56
PEG-BHD1028 25ug/Kg (g)	5.71 ±0.31	9.08 ±0.90	9.70 ±0.65	9.39 ±0.34	8.68 ±0.55	7.97 ±0.43	7.94 ±0.31	8.92 ±0.36	8.42 ±0.48
PEG-BHD1028 50ug/Kg (g)	5.71 ±0.44	8.45 ±0.24	9.00 ±0.37	9.35 ±0.48	9.81 ±0.43	9.45 ±0.31	7.92 ±0.41	8.55 ±0.30	8.53 ±0.37
PEG-BHD1028 200ug/Kg (g)	5.34 ±0.24	8.11 ±0.66	8.42 ±0.62	8.82 ±0.53	9.52 ±0.52	7.75 ±0.35	7.46 ±0.15	7.63 ±0.10	7.88 ±0.40
